# *QuickStats:* Percentage* of Adults Aged ≥18 Years Who Have Ever Had Hepatitis,^†^ by Age Group and Sex — National Health Interview Survey,^§^ United States, 2021

**DOI:** 10.15585/mmwr.mm7202a6

**Published:** 2023-01-13

**Figure:**
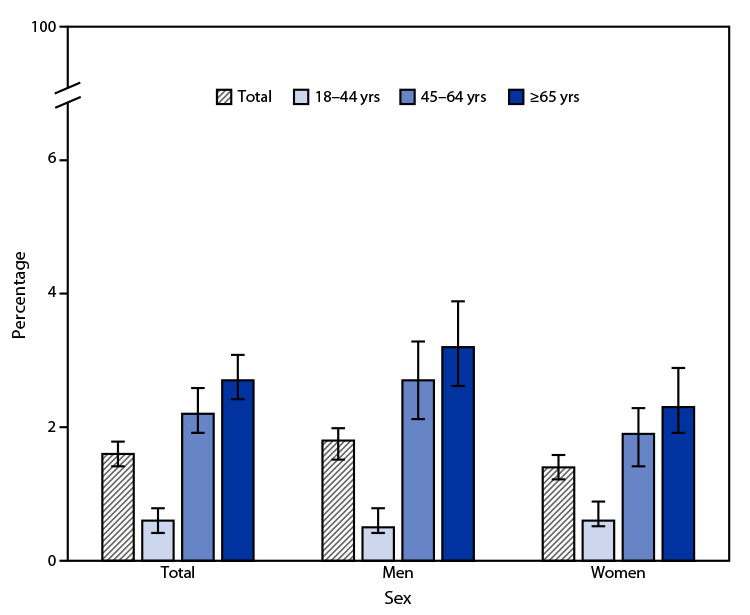
In 2021, 1.6% of adults aged ≥18 years reported having ever had hepatitis. The prevalence of hepatitis was lowest among adults aged 18–44 years (0.6%) and highest among adults aged ≥65 years (2.7%). Prevalence increased with age for both men and women. The percentage of adults who ever had hepatitis was higher in men than women aged 45–64 years (2.7% versus 1.9%) and ≥65 years (3.2% versus 2.3%) but was similar in adults aged 18–44 years (0.5% versus 0.6%).

**Source**: National Center for Health Statistics, National Health Interview Survey, 2021. https://www.cdc.gov/nchs/nhis.htm

